# Three Classes of Antioxidant Defense Systems and the Development of Postmenopausal Osteoporosis

**DOI:** 10.3389/fphys.2022.840293

**Published:** 2022-03-03

**Authors:** Keda Yang, Fangming Cao, Yuchuan Xue, Lin Tao, Yue Zhu

**Affiliations:** ^1^Department of Orthopedics, First Hospital of China Medical University, Shenyang, China; ^2^The First Department of Clinical Medicine, China Medical University, Shenyang, China

**Keywords:** postmenopausal osteoporosis, oxidative stress, antioxidant system, PI3K/AKT/Nrf2/HO-1, GSH/GSSG

## Abstract

Osteoporosis is a common bone imbalance disease that threatens the health of postmenopausal women. Estrogen deficiency accelerates the aging of women. Oxidative stress damage is regarded as the main pathogenesis of postmenopausal osteoporosis. The accumulation of reactive oxygen species in the bone microenvironment plays a role in osteoblast and osteoclast apoptosis. Improving the oxidative state is essential for the prevention and treatment of postmenopausal osteoporosis. There are three classes of antioxidant defense systems in the body to eliminate free radicals and peroxides including antioxidant substances, antioxidant enzymes, and repair enzymes. In our review, we demonstrated the mechanism of antioxidants and their effect on bone metabolism in detail. We concluded that glutathione/oxidized glutathione (GSH/GSSG) conversion involved the PI3K/Akt-Nrf2/HO-1 signaling pathway and that the antioxidant enzyme-mediated mitochondrial apoptosis pathway of osteoblasts was necessary for the development of postmenopausal osteoporosis. Since the current therapeutic effects of targeting bone cells are not significant, improving the systemic peroxidation state and then regulating bone homeostasis will be a new method for the treatment of postmenopausal osteoporosis.

## Introduction

Osteoporosis is a metabolic bone disease characterized by a decrease in bone mass per unit volume. Elderly and postmenopausal women are at high risk of osteoporosis (Srivastava and Deal, 2002). The thin cortical bone and sparse cancellous bone increase the risk of fractures in patients with osteoporosis, which seriously threaten public health and cause a huge social burden (Black and Rosen, 2016). At present, more studies have focused on osteoblasts and osteoclasts. Most of the drugs used to treat osteoporosis directly act on the process of bone formation and absorption, mainly inhibiting osteoclasts. Osteogenesis drugs are parathyroid hormone (PTH), prostaglandin E2 (PGE2), calcium, and vitamin D (Kabasawa et al., 2003). Drugs that inhibit osteoclasts include estrogen replacement treatment and bisphosphonates (Qaseem et al., 2017). Improving lifestyle and eating habits also helped prevent osteoporosis. These treatments are not fully satisfying due to limitations in bone microenvironmental regulation. They ignore the complex changes in the body caused by estrogen deficiency. Estrogen or selective estrogen receptor modulators have a better effect, although there are some limitations in indications (Mandelli et al., 2021). Therefore, to propose a more effective and widely applicable treatment method, fully analyzing the pathogenesis of postmenopausal osteoporosis and the pathological changes of the body is a necessary means.

**FIGURE 1:**
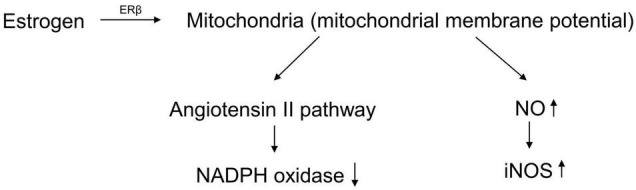
The antioxidant effect of estrogen.

Recent studies have shown that the pathogenesis of postmenopausal osteoporosis is mainly due to aging (Pignolo et al., 2021). Aging is believed to be caused by the accumulation of reactive oxygen species (ROS) (Davalli et al., 2016). ROS are generated by various organelles, especially mitochondria, through enzymatic and non-enzymatic reactions in cell metabolism (Zorov et al., 2014). ROS can cause DNA damage and protein denaturation, thereby causing gene mutations and affecting normal biological functions (Zhang et al., 2020a). Additionally, some ROS, such as oxygen-containing free radicals, are also regarded as inflammatory mediators that affect the microenvironment of the organism, leading to the occurrence of diseases (Forrester et al., 2018). Under physiological conditions, there are three classes of antioxidant defense systems in the human body to remove excess ROS and avoid oxidative damage, including antioxidant substances, antioxidant enzymes, and repair enzymes. In the detection of blood oxidative stress indicators in postmenopausal women, it was found that antioxidant indicators, including glutathione peroxidase (GSH-Px), folate, and superoxide dismutase, decreased significantly (Zhou et al., 2016). Estrogen could protect mitochondrial membrane potential through estrogen receptor beta (ERβ) (Simpkins et al., 2008). The antioxidant effect depended on decreasing the activity of NADPH oxidase *via* the angiotensin II (Ang II) pathway and reducing inducible nitric oxide synthase (iNOS) by enhancing NO activity (Miyazaki-Akita et al., 2007; Yung et al., 2011; Figure 1). Dysfunction of the antioxidant defense systems causes redox imbalance and leads to the body being in a state of peroxidation, which makes it difficult to remove ROS.

As mentioned above, the weakening of the body’s antioxidant capacity, leading to the accumulation of free radicals and inducing bone aging, is a necessary cause of postmenopausal osteoporosis. Improving antioxidant capacity and removing excess ROS will be an effective method for the systemic treatment of osteoporosis. Therefore, we reviewed the relationship between the three classes of antioxidant systems and the development of postmenopausal osteoporosis.

## Reactive Oxygen Species in Postmenopausal Osteoporosis

**FIGURE 2:**
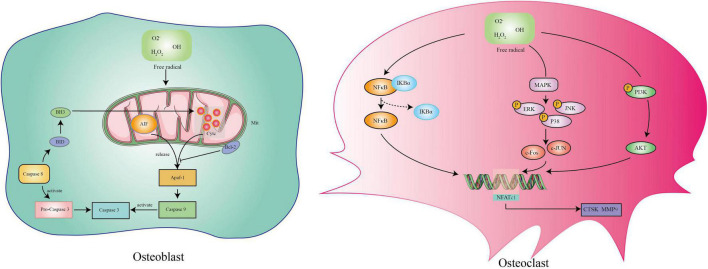
The mechanism of reactive oxygen species on osteoblasts and osteoclasts.

The essence of postmenopausal osteoporosis is weakened osteogenesis and increased osteoclastogenesis caused by the lack of estrogen. However, the pathogenesis remains unclear. With continuous exploration, researchers have paid more attention to aging accelerated by estrogen deficiency and have shown that oxidative stress damage is the pathogenesis of postmenopausal osteoporosis (Mohamad et al., 2020; Tu et al., 2020; Shahriarpour et al., 2021). The accumulation of ROS is regarded as an important factor in destroying bone homeostasis without estrogen protection (Mohamad et al., 2020). On the one hand, estrogen activates endothelium-derived hyperpolarizing factor (EDHF) to release NO and modulates NADPH oxidase involved in the Ang II process to inhibit ROS production in skeletal vascular endothelium (Silva, 2021; Youn et al., 2021). On the other hand, estrogen upregulates MnSOD activity and inhibits cellular ROS production (Oh et al., 2019). The main effect of ROS on osteoblasts is to induce the cell mitochondrial apoptosis pathway (Luo et al., 2021). ROS change the permeability of mitochondrial membranes and release internal apoptotic factors including cytochrome c (Cytc) and apoptosis-inducing factor (AIF) (Zhao et al., 2020a; Seminotti et al., 2021). These factors combine with apoptotic protease activating factor-1 (Apaf-1) and activate caspase-9 and caspase-3 in the cytoplasm, causing cell apoptosis (Wang et al., 2019). The positive effect of ROS on osteoclasts is to promote differentiation. ROS can activate three essential pathways involved in osteoclast differentiation including the MAPK, PI3K, and nuclear factor kappa-B (NF-κB) pathways (Thummuri et al., 2017; Zhou et al., 2020; Xiao et al., 2021). The activation of these pathways contributes to the expression of the osteoclast maturation genes CTSK, MMP9, and NFATC1 (Tao et al., 2020). In conclusion, there is an axis of estrogen deficiency/ROS accumulation/osteoblast apoptosis and osteoclast differentiation in postmenopausal osteoporosis (Figure 2).

## Antioxidant Substances

### Glutathione

The biosynthesis of glutathione (GSH) mainly reduces oxidized glutathione (GSSG) with glutathione reductase, assisted by NADPH produced by the pentose phosphate pathway (Fan et al., 2014). The sulfhydryl group (-SH) in GSH provides reducing hydrogen to give free radicals a pair of electrons, so that the free radicals lose their strong oxidizing and aggressive properties (Bánhegyi et al., 2003). Previous studies have shown that serum GSH levels were significantly reduced in osteoporotic rats (Yalin et al., 2012; Ameen et al., 2020). Increasing the GSH/GSSG ratio can attenuate the oxidative damage of ROS on osteoblasts *via* the PI3K/Akt-Nrf2 signaling pathways (Casati et al., 2020). Nrf2 is a nuclear factor that regulates gene encoding proteins involved in the response to injury and inflammation, including the production of free radicals (Süntar et al., 2021). When the PI3K/AKT pathway located on the cell membrane is activated, the signal is transmitted to the cytoplasm to release Nrf2 anchored by Kelch-like ECH-associated protein 1 (Keap1) (Chen et al., 2021a). After Nrf2 enters the nucleus, it forms a coactivator complex with the small Maf protein. This heterodimer binds to the promoter region of the antioxidant response element (ARE) to activate the expression of antioxidant genes (Shan et al., 2021). It has also been demonstrated that the activation of Nrf2 promotes osteogenic differentiation by increasing heme oxygenase-1 (HO-1) expression (Liu et al., 2018; Chen et al., 2020b). However, it is worth noting that the overexpression of Nrf2 might inhibit the differentiation of osteoblasts (Chen et al., 2021b). GSH inhibits the osteoclast differentiation-mediated NF-κB signaling pathway induced by ROS (Han et al., 2020). Nrf2 is considered to be a key factor in alleviating the formation of osteoclasts in inflammatory bone loss (Hong et al., 2021). Nrf2 also modulates NFATc1, the main transcription factor secreted by osteoclasts, to inhibit osteoclast differentiation (Sun et al., 2015). Therefore, the GSH/Nrf2-mediated antioxidant pathway is essential for the balance of osteogenesis and osteoclastogenesis.

### Vitamin C and Vitamin E

Vitamin C is an antioxidant that can protect -SH and keep the -SH of sulfhydrylase in a reduced state. It reduces GSSG to GSH to remove lipid oxides from cell membranes with the assistance of glutathione reductase (Xu et al., 2020). Vitamin C, also known as ascorbic acid, is an important bone-promoting substance (Mizerska-Kowalska et al., 2019). It can be combined with β-glycerophosphate sodium and dexamethasone for the differentiation induction of osteoblasts (Dey et al., 2020). On the one hand, vitamin C can promote the expression of osteogenic genes including bone morphogenetic protein-2 (BMP2) and runt-related transcription factor 2 (Runx2) (Choi et al., 2019). On the other hand, it assists proline hydroxylase in promoting the maturation of collagen and the production of osteocalcin (OCN) in the bone matrix (Chojkier et al., 1983; Nielsen-Marsh et al., 2005).

The physiological function is mainly to resist free radicals produced by lipid peroxidation on biological membranes. However, the mechanism of vitamin E is to capture lipid peroxide free radicals and then to reduce them by glutathione or vitamin C, instead of directly acting as a reducing substance. For the study of vitamin E in the skeletal system, Vakili et al. (2021) found that vitamin E can improve bone mass in ovariectomized rats by inhibiting bone cell apoptosis and autophagy. However, compared with osteoblasts, vitamin E has a stronger inhibitory effect on osteoclasts. Receptor activator of NF-κB (RANK), receptor activator of NF-κB ligand (RANKL), osteoprotegerin (OPG), and Wnt/β-catenin signaling were involved in the process of inhibiting osteoclastogenesis (Wong et al., 2019). In addition to its direct effects on the skeletal system, vitamin E is also involved in inflammatory and immune responses and intervenes in bone metabolism by regulating bone-resorbing cytokines including interleukin-1 (IL-1) and IL-6 (Nazrun et al., 2012; Nazrun Shuid et al., 2019).

In a clinical trial, researchers administered vitamin C and E alone or in combination with patients with osteoporosis and found that the bone mass of the patients was significantly improved, and the serum antioxidant level was significantly increased (Chavan et al., 2007), which indicated that vitamins C and E are effective in the treatment of osteoporosis.

### Melatonin

Melatonin is an endogenous antioxidant hormone secreted by the pineal gland. Melatonin can directly combine with reactive oxygen free radicals and reactive nitrogen-free radicals (Zhao et al., 2008). The combined product is chemically stable, and free radicals combined with melatonin cannot be regenerated. Our previous study revealed that intragastric melatonin could significantly improve bone mass in postmenopausal mice (Da et al., 2020). Melatonin could enhance osteogenic effects by increasing SIRT1 and SIRT3, the essential factors regulating antioxidant enzyme formation in mitochondria (Qiu et al., 2019; Zhou et al., 2019; Chen et al., 2020b). Melatonin also directly prevents ROS damage by eliminating lipid peroxide and lipopolysaccharide (LPS) (Yu and Tan, 2019; Hossain et al., 2020). In addition to alleviating oxidative stress damage, melatonin affected bone homeostasis by regulating the rhythm of the biological clock (Song et al., 2018). OCN and type I collagen (collagen I) showed a strong correlation with the melatonin rhythm, leading to bone remodeling destruction when circadian disturbances occurred, which was demonstrated in postmenopausal women (Munmun and Witt-Enderby, 2021). The direct relationship between melatonin and bone metabolism is closely related. Melatonin could induce osteogenic differentiation *via* the BMP/Wnt signaling pathway (Park et al., 2011). BMP proteins regulate the recruitment and activation of Smad family transcription factors (Zhao et al., 2020b). Then, the Wnt/β-catenin signaling pathway could be activated by upstream signal (Wang et al., 2020). β-catenin could promote the expression of osteogenic factors including Runx2, osterix, and type I collagen (Oh et al., 2021). At present, there are many clinical studies using melatonin as an auxiliary drug for the treatment of osteoporosis.

### Protein Antioxidants: Ferritin and Ceruloplasmin

Fe2+ is very active and can react with oxygen to produce hydroxyl radicals and peroxide radicals and become Fe^3+^ with strong oxidizing properties (Obraztsov et al., 1975). Ferritin combines with Fe3+ to store iron and to relieve the oxidative damage of iron ions (Zhao et al., 2006). Ferritin is closely related to the development of postmenopausal osteoporosis. A serum test of 4,000 women found that serum ferritin is more closely related to bone density than iron intake and serum iron, which indicates that ferritin is a more reliable variable linking iron and osteoporosis (Lu et al., 2020). M. Spanner demonstrated that ferritin was widely expressed in osteoblastic lineage cells to maintain the intracellular metal balance through the uptake and storage of iron (Spanner et al., 1995). The positive effect of ferritin on osteoporosis is mainly to inhibit iron ions. Iron and iron-induced ROS accumulation was indicated to mediate osteoblast apoptosis and osteoclast differentiation *via* the NF-κB signaling pathway (Wang et al., 2018; Liu et al., 2021). Increasing the combination of ferritin and iron ions is an effective means to relieve iron damage and ferritin to treat osteoporosis.

Ceruloplasmin (CER), also called copper oxidase, is an important antioxidant in the body. Its antioxidant effect mainly inhibits the production of ROS induced by Fe^2 + 73^. CER can reduce free radicals produced in xanthine metabolism by inhibiting xanthine oxidase (Krsek-Staples and Webster, 1993). Similar to ferritin, the effect of inducing lipid peroxidation by copper ions has also been weakened when combined with CER (Burkitt, 2001). Current research indicates that CER relieves osteoporosis by inhibiting iron overload (Zarjou et al., 2010). However, the direct effect of CER on osteoporosis is unclear.

## Antioxidant Enzymes

### Superoxide Oxidoreductase

Superoxide oxidoreductase (SOD), an endogenous antioxidant metalloenzyme, is the first defense system of antioxidant enzymes that can catalyze the dismutation reaction of superoxide radical anion ($•O_{2}^{-}$) to transform into hydrogen peroxide (H_2_O_2_) (Lushchak, 2014). A previous study indicated that the total SOD level decreased in menopausal mice, as measured by ELISA (Cao et al., 2020). Hidetoshi Nojiri found that bone mass decreased significantly in SOD-deficient mice (Nojiri et al., 2011). SOD activity is regulated by Smad family transcription factors and is directly involved in the OPG/RANKL/RANK axis to maintain bone homeostasis by increasing osteoblast and inhibiting osteoclast differentiation (Lee et al., 2010; Araújo et al., 2017; Jiang et al., 2020; Qi et al., 2021). Increasing mitochondrial SOD activity prevented osteoblast apoptosis induced by ROS (Yang et al., 2021a). Moreover, SOD has a significant regulatory effect on the differentiation trend of bone marrow mesenchymal stem cells (BM-MSCs). SOD participates in the osteogenesis-adipogenesis balance of BM-MSCs by inhibiting ROS impairment *via* the p38/MARK pathway (Jurczuk et al., 2004; Wang et al., 2017).

### Catalase

Catalase (CAT) is the marker enzyme of peroxisomes and is widely present in various tissues of the body (Shin et al., 2018). As the second defense system of antioxidant enzymes, the antioxidant mechanism of CAT mainly acts on the dismutation reaction on H_2_O_2_ produced in SOD-mediated processes (Ray and Husain, 2002). A large number of studies have reported that H_2_O_2_ increased lipid peroxidation following the decrease of CAT in a postmenopausal osteoporosis model (Ozgocmen et al., 2007; Sendur et al., 2009; Zhou et al., 2016). The main effect of CAT is promoting osteogenic differentiation *via* the Nrf2/HO-1 pathway and preventing mitochondrial apoptosis of osteoblasts as mentioned above (Mada et al., 2017; Sant et al., 2018). In addition, CAT can have a positive effect on bone mass by inhibiting H_2_O_2_-induced osteoclastic resorption (Fraser et al., 1996). However, the physiological role of CAT is mainly dependent on the regulation of forkhead box O1 (FOXO1) (Venkatesan et al., 2007). FOXO1 increased the expression of SIRT1 participating in the mitochondrial biosynthesis to maintain the level of CAT (Alcendor et al., 2007). Estrogen deficiency decreases the expression of FOXO1 protein, leading to the inhibition of BM-MSCs into osteoblasts (Liao et al., 2016). Therefore, the FOXO1/CAT pathway has a potential effect on osteoporosis treatment.

### Glutathione Peroxidase

Glutathione peroxidase (GSH-Px) is an important peroxidase enzyme characterized by each subunit containing a selenium (Se) atom in the form of selenocysteine (Liu et al., 1999). The antioxidant effect of GSH-Px is determined by the Se cysteine in its active center (Zachara, 1992). The Se of the GSH-Px enzyme system catalyzes GSH to GSSG and reduces toxic peroxides to non-toxic hydroxyl compounds. GSH-Px can intervene in the development of osteoporosis through the abovementioned GSH-dependent pathways and endoplasmic reticulum-mediated osteogenic differentiation of BM-MSCs *via* the mTOR pathway (Wiswedel et al., 2010; Hu et al., 2021). Additionally, GSH-Px can relieve inflammation-induced osteolytic bone destruction by breaking down LPS (Islam et al., 2007; Li et al., 2020). Upregulating GSH-PX activity can inhibit pro-inflammatory factors associated with osteoclast maturation genes, such as iNOS, IL-1β, and tumor necrosis factor-alpha (TNF-α) (Kruger et al., 2010; He et al., 2020; Li et al., 2020; Umar et al., 2021). GSH-Px may be the key link in the oxidative stress-inflammation reaction in postmenopausal osteoporosis with great potential research value. However, the content of peroxidase in the fracture site was increased to compensate for fracture-induced stress damage when fractures occurred in patients with osteoporosis (Föger-Samwald et al., 2016).

## Repair Enzymes

### DNA Repair: Glycosylase, AP-Endonuclease, and DNA Polymerase

The metabolism of free purine bases after DNA damage aggravates oxidative stress damage. This process continues to convert O_2_ into $•O_{2}^{-}$, H_2_O_2_, and •OH, enhancing oxidative damage. In contrast, a growing number of studies have shown that high uric acid levels can lead to decreased bone density and osteoporosis (Sharaf El Din et al., 2017). The ROS produced by the oxidation of purine inhibits osteoblast differentiation from BM-MSCs and bone mineralization through the ERK and NF-KB pathways (Chang et al., 2009). ROS can stimulate the proliferation and differentiation of osteoclast progenitor cells through the RANKL pathway (Garrett et al., 1990). In addition to direct effects, purine metabolism also regulates bone homeostasis through the indirect activation of inflammatory cytokines (Martinon, 2010). At high uric acid levels, mononuclear cell-derived inflammasomes phagocytose monosodium urate (MSU) and release IL-1, TNF-α, IL-6, and IL-8 (Chhana et al., 2018). They further activate RANK and macrophage colony-stimulating factor (M-CSF), resulting in a large number of osteoclasts (Ritchlin et al., 2003). Therefore, the damaged DNA must be repaired to ensure genomic integrity (Radak and Boldogh, 2010). DNA repair enzymes reduce the production of purine bases to prevent further damage to the skeletal system, as well as the occurrence of osteoporosis (Yao et al., 2020).

### Lipid Peroxide Metabolism: Phospholipase A2 and Acyltransferase

**FIGURE 3:**
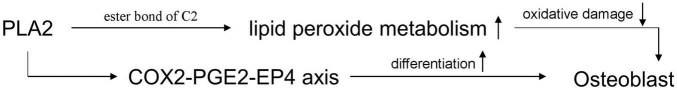
The mechanism of PLA2 on increasing osteogenesis.

Lipid peroxide, a peroxidation product of unsaturated fatty acids with ROS, is the core of lipofuscin (Adibhatla and Hatcher, 2008; Zadlo et al., 2017). The accumulation of lipid peroxide will disrupt the body’s acid balance and vitamin utilization, leading to faster cell division and aging (Zhang et al., 2020b). Previous studies indicated that a large amount of lipid peroxide is deposited in the bone tissue of ovariectomized mice (Al et al., 2018; Abdallah et al., 2020). It triggers oxidative damage and inflammation in the bone microenvironment, destroying bone homeostasis (Wu et al., 2016; Li et al., 2018). Oxidized lipids cannot be repaired and need to be broken down into non-toxic products by specific enzymes. Phospholipase A2 (PLA2) and acyltransferase (AT) are the important metabolic enzymes of lipid peroxide. AT directly participates in lipid mobilization and β-oxidation (Wang et al., 2021). PLA2 catalyzes the hydrolysis of the ester bond formed by the C2 hydroxyl group on the glycerol backbone in the phospholipid (Prunonosa Cervera et al., 2021). Compared with AT, the effect of PLA2 on the skeletal system is not limited to accelerate the metabolism of lipid peroxide. PLA2 can increase the expression of PGE2 to promote osteogenesis through the cyclooxygenase 2 (COX2) pathway (Yoshida et al., 2007; Piazzolla et al., 2015; Li et al., 2021; Figure 3).

## Discussion

At present, osteoporosis affects approximately one-third of postmenopausal women worldwide (Gosset et al., 2021). Nearly 50% of these women will develop osteoporosis-related fractures (Porter and Varacallo, 2021). In the past, scientific researchers and clinicians focused on the skeletal system, especially the inhibition of osteoclasts, to treat postmenopausal osteoporosis (Ukon et al., 2019; Hsiao et al., 2020). Although the deterioration has been improved to a certain extent, the pathogenesis of the disease has not been clarified, and effective control has not been achieved. Oxidative stress damage, as a mediator linking estrogen, aging, and bone, is regarded as a breakthrough in exploring the development of postmenopausal osteoporosis. Overloaded ROS break the balance between osteogenesis and osteoclastogenesis, leading to bone mass loss and bone quality decline (Lee et al., 2021).

**FIGURE 4:**
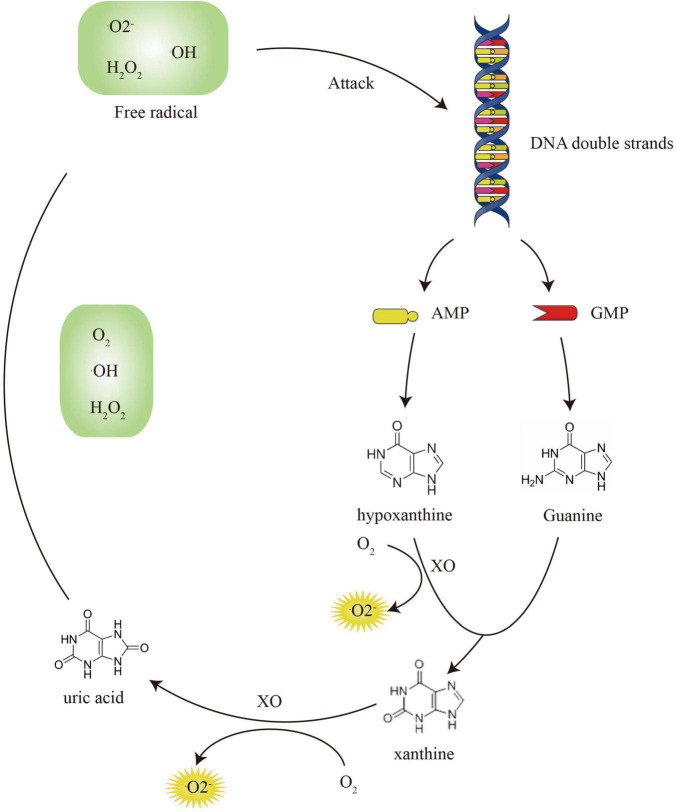
The vicious circle of DNA damage and purine metabolism.

**FIGURE 5:**
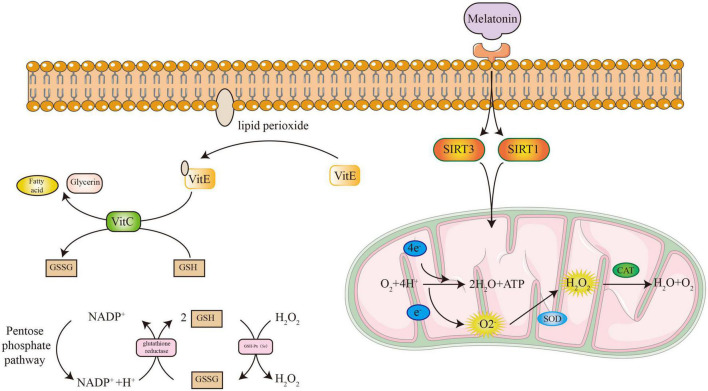
The process of antioxidant systems to eliminate free radicals.

The ROS accumulation is due to excessive production and inefficient removal. The DNA damage caused by ROS and the metabolism of DNA purine bases form a closed loop, which continuously increases the production of free radicals (Calkins et al., 2016; Taras-Goslinska et al., 2019). DNA repair enzymes are essential to break this vicious cycle (Figure 4). For the elimination of ROS shown in Figure 5, antioxidant enzymes and GSH/GSSH conversion play an essential role in mitochondria (Zhang et al., 2021). The endogenous hormone melatonin maintains these antioxidant processes by protecting mitochondrial function (Yang et al., 2021b). Other antioxidant substances, such as protein and vitamin antioxidants, also assist the conversion of GSH/GSSG to provide electrons to free radicals to alleviate oxidative damage. The protective effect of the GSH/Nrf2 pathway and SOD-CAT on mitochondrial apoptosis contributes to osteogenesis. The ROS activation of the NF-κB pathway involved in osteoclast maturation is also inhibited by antioxidant systems.

Inflammation is also an important connection between oxidative stress and postmenopausal osteoporosis. Oxidative stress causes biomolecular damage and releases cytokines and chemokines to recruit and activate inflammatory cells, resulting in chronic inflammation in the body (Sindhu et al., 2020). ROS can induce the hyperactivation of NF-κB by modulating the activity of AP-1 (Arcambal et al., 2019). Activated NF-κB also induces the expression of inflammatory factors, such as IL-1β, IL-6, and TNF-α to exacerbate inflammation (Ma et al., 2020). These inflammatory factors also stimulate ROS production to exacerbate oxidative damage (Zhu et al., 2022). A vicious cycle exists between oxidative stress and inflammation. Osteoporosis is also regarded as a chronic inflammatory disease (Montalcini et al., 2013). Estrogen deficiency could induce an inflammatory storm and decrease antioxidant capacity (Mohamad et al., 2020). The secretion of inflammatory factors activates osteoclasts to worsen osteoporosis (Wu et al., 2020; Pan et al., 2021). Therefore, anti-inflammatory drugs have been applied to treat osteoporosis and have the ability to improve bone mass (Tao et al., 2021; Feng et al., 2022).

Due to the unclear pathogenesis of postmenopausal osteoporosis, past studies have obvious limitations. Our review clarified the nature of postmenopausal osteoporosis from the perspective of oxidative stress damage induced by aging and described the potential ability of antioxidants to treat it in detail. Antioxidants not only systematically improve the oxidation state of the body, but also locally regulate the imbalance of the skeletal system. At present, antioxidant substances have been verified to improve bone mass in animal models, such as vitamin C, vitamin E, and GSH (Deng et al., 2014; Lindsey et al., 2019; Han et al., 2020). However, there is no special drug designed based on the antioxidant ability that is being applied for osteoporosis treatment. Three classes of antioxidant systems are very important for the prevention and treatment of postmenopausal osteoporosis. Our review contributes to antioxidant drug designs for postmenopausal osteoporosis.

## Author Contributions

KY contributed to data curation, formal analysis, data curation, methodology, and writing – original draft. FC contributed to investigation, methodology, software, and writing – original draft. YX contributed to software and validation. LT contributed to conceptualization, software, validation, writing, review, and editing. YZ contributed to funding acquisition, project administration, resources, writing, reviewing, and editing. All authors read and approved the manuscript.

## Conflict of Interest

The authors declare that the research was conducted in the absence of any commercial or financial relationships that could be construed as a potential conflict of interest.

## Publisher’s Note

All claims expressed in this article are solely those of the authors and do not necessarily represent those of their affiliated organizations, or those of the publisher, the editors and the reviewers. Any product that may be evaluated in this article, or claim that may be made by its manufacturer, is not guaranteed or endorsed by the publisher.
